# The Protective Effects of Silver Fluoride Solution and Fluoride Varnish on Dental Erosion—An In Vivo Study

**DOI:** 10.3390/dj13020046

**Published:** 2025-01-22

**Authors:** Julie Marie Haabeth Brox, Amela Tulek, Amer Sehic, Aida Mulic, Tor Paaske Utheim, Qalbi Khan

**Affiliations:** 1 Department of Oral Biology, Faculty of Dentistry, University of Oslo, Postboks 1052 Blindern, 0316 Oslo, Norway; j.m.h.brox@odont.uio.no (J.M.H.B.); uxutto@ous-hf.no (T.P.U.); qalbi.khan@odont.uio.no (Q.K.); 2 Nordic Institute of Dental Materials (NIOM AS), Sognsveien 70A, 0855 Oslo, Norway; amela.tulek@niom.no (A.T.); aida.mulic@niom.no (A.M.); 3 Department of Maxillofacial Surgery, Oslo University Hospital, Postboks 4950 Nydalen, 0424 Oslo, Norway; 4 Department of Clinical Dentistry, Faculty of Health Sciences, UiT The Arctic University of Norway, 9037 Tromsø, Norway; 5 Department of Medical Biochemistry, Oslo University Hospital, 0424 Oslo, Norway; 6 Department of Plastic and Reconstructive Surgery, Oslo University Hospital, 0424 Oslo, Norway; 7 Department of Ophthalmology, Sørlandet Hospital Arendal, 4838 Arendal, Norway; 8 Department of Public Health and Sport Sciences, Inland Norway University of Applied Sciences, Hamarvegen 112, 2406 Elverum, Norway

**Keywords:** dental erosion, silver diamine fluoride, fluoride varnish, preventive treatment, dentin hypersensitivity

## Abstract

**Objectives:** Dental erosion has evolved into a common condition with growing concern in the dental community. The aim of this study was to explore the protective effects of two highly fluoridated products, silver fluoride solution (silver diamine fluoride-potassium iodide solution, SDF-KI) and fluoride varnish, on dental erosion in mouse. **Methods:** Two groups of ten young CD-1 mouse were given a cola drink ad libitum over a 6-week period. A prophylactic treatment with a silver fluoride solution (38% SDF 48,000 ppm F with a silver concentration of 253,900 ppm) and a fluoride varnish (NaF, 22,600 ppm F) were applied on the mandibular molars, performed under sedation twice a week during the whole experiment. Furthermore, two control groups of ten mice were included, a positive (cola drink) and a negative (distilled water) control. A terminal procedure was followed by dissection of mandibular molars and analysis of them using scanning electron microscopy (SEM). The first molars were transversely ground, further analyzed by SEM, and measured for tooth height and tooth tissue loss. **Results:** Analyses of mandibular molars treated with a fluoride varnish indicated a 5% decreased tooth tissue loss, compared to the molars which served as a positive control. The best effect was achieved with the application of silver fluoride solution, displaying a 35% decreased tooth tissue loss compared to the positive control group. **Conclusions:** Preventive treatment with a solution of silver fluoride solution exhibits greater protection against dental erosion compared to a traditional fluoride varnish. This study indicates that silver fluoride solution is an effective fluoride compound and is highly beneficial in a clinical setting with the aim of preventing dental erosion.

## 1. Introduction

Dental erosion is an increasing problem globally, with a reported prevalence from 20% to 45% in permanent teeth [1]. The primary cause of dental erosion arises from intrinsic and extrinsic factors, i.e., the oral cavity being repeatedly exposed to acids such as gastric acid and various acids originating from acidic drinks or food [2]. With time, the presence of these acids may cause permanent, irreversible loss of the dental hard tissue. Moreover, the modern lifestyle, with frequent consumption of acidic drinks, has a manifold influence on the body. Excessive consumption of soft drinks is considered a determining factor of the development of diabetes [3] and obesity [4].

A recent analysis exploring food sales in Norwegian grocery stores discovered that the most frequently bought item was carbonated and non-carbonated acidic drinks [5]. In the opposite direction, a trend in the Norwegian diet showed a massive decline in the consumption of milk [6]. With a decreased intake of milk, the chance of calcium deficiency increases, hence affecting our teeth and bones and threatening the build-up of maximal peak bone mass at a vital time in life [7]. In fact, the addition of calcium to an acidic drink has shown results of reducing enamel erosion [8]. Furthermore, the findings suggest that physical activity is associated with a greater risk of developing dental erosion, notably due to frequent intake of sports drinks [9]. Recurring symptoms associated with dental erosion are dentin hypersensitivity, imbalanced occlusion affecting function, and impaired esthetics [10]. Being an irreversible process, dental erosion treatment can be demanding, expensive, and complex [11,12]. There has been an increased focus on minimally invasive dentistry, which aims to preserve the natural tooth and pulp vitality. The importance of and interest in preventive treatment with oral health education, dietary analysis, and the use of anti-erosive agents are also on the rise [13].

Fluoride is considered an effective anti-erosion agent, being either monovalent fluoride or polyvalent fluoride [14,15]. The protective effect of fluoride is achieved by forming a protective layer of calcium fluoride (CaF_2_) deposits, forming fluorapatite on the tooth surface, and modifying the proteins in the salivary pellicle [15,16,17]. Silver diamine fluoride (SDF, 38% SDF 48,000 ppm F and a silver concentration of 253,900ppm) is a polyvalent fluoride with metal cations that is considered safe and effective for oral disease treatment [18]. Preliminary studies predicted that SDF forms a protective barrier consisting of a calcium fluoride-like layer (CaF_2_), silver chloride (AgCl), and metallic silver and forms fluorapatite (FAP) crystals [19]. In addition, recent studies have found an important effect of SDF being an inhibitor of matrix metalloproteinases (MMPs) and thereby preserving the collagen network in the dentin [13,20]. Even though the application of SDF poses a risk of tooth surface staining, this is supposed to be reduced with the application of potassium iodide (KI) directly after SDF. The result is scavenging the free silver ions, hence creating a creamy white-silver iodide precipitate [21].

Fluoride varnish (NaF, 22,600 ppm F) has a fast setting time and a prolonged adherence to the tooth surface, making the fluoride release slow and efficient [22]. In this way, fluoride varnish is thought to promote more CaF_2_ precipitation on the tooth surface over time [22]. Even though this CaF_2_ layer dissolves when exposed to acid, it functions as a physical barrier, hampering the dissolution of the underlying tooth mineral [23]. Nonetheless, Carvalho et al., 2015, proposed that the protective effects of fluoride varnish were mostly related to the adherence to the tooth by the resins, forming a mechanical barrier which is easily worn down [24].

Human studies on dental erosion raise ethical concerns due to the permanent loss of dental hard tissue. Our research group has developed a mouse model designed for inducing extrinsic dental erosion, whereby varying severity of dental lesions can be generated and analyzed [25]. This method includes the examination of transversely ground molars utilizing scanning electron microscopy (SEM) to register dental erosion and its severity, despite the study of miniature dentition in the form of mouse molars [25].

The aim of the current in vivo study was to analyze and compare the protective strength of a solution of silver fluoride with fluoride varnish when generating dental erosion using the established mouse model. The null hypothesis states that there is no difference in the preventive effects of silver fluoride solution and fluoride varnish on dental erosion. To our knowledge, this is the first in vivo study to examine and compare the effects of these fluoride compounds for prophylactic treatment of dental erosion.

## 2. Materials and Methods

### 2.1. Animal Model

Included in this study were forty phenotypically young female mice (CD-1 strain, 7 weeks old, 30 ± 5 g body weight). The mice were bred and the study was conducted at the department for comparative medicine, Faculty of Medicine, University of Oslo. To reduce the rate of erosive tooth wear (ETW), several measures were implemented. Wire cages with a solid bottom and bedding were arranged in the correct manner to reduce the wear of the dentition. Hard objects such as wooden sticks and plastic wheels were removed from the cages, with paper boxes and paper ribbons serving as part of environmental enrichment. The experiment followed Norwegian regulations and legislation (Norwegian Regulation on Animals Used for Scientific Purposes 2010/63/EU and the Norwegian Animal Welfare Act of 2009). The current study was authorized by the Norwegian Food Safety Authority (FOTS ID 28734), date of approval 22 November 2021.

**Figure 1:**
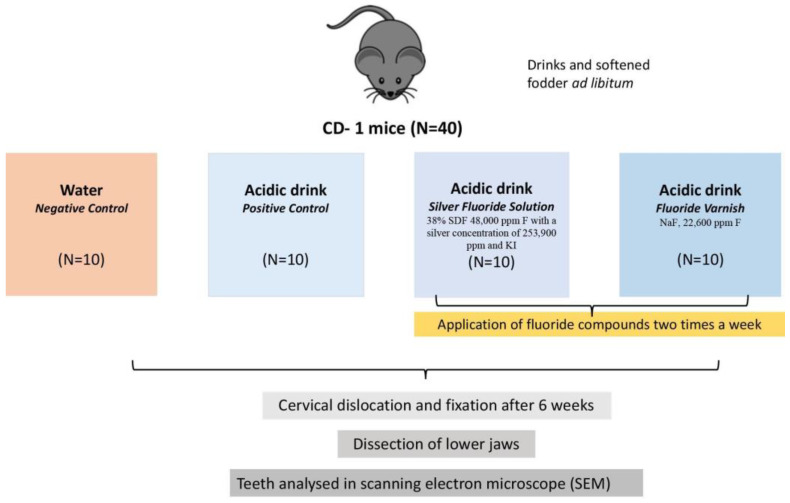
A flowchart of the study animal’s enrollment. A total of 40 mice were assigned to four experimental groups. Three groups were given an acidic beverage (Coca Cola, phosphoric acid, pH = 2.27), while the fourth group served as a negative control, receiving distilled water. Of the three experimental groups, two were treated with topical agents applied to the molar teeth: one group received a silver fluoride solution containing silver diamine fluoride (38% SDF, 48,000 ppm F, 253,900 ppm silver) followed by potassium iodide (KI) and the second group received fluoride varnish (NaF, 22,600 ppm F). After six weeks, all animals were euthanized via cervical dislocation, and their teeth were prepared for SEM analysis.

Forty mice were randomly assigned to four groups, i.e., ten animals in each group (Figure 1). Three experimental groups were given an acidic drink (Coca-Cola, phosphoric acid, pH = 2.27), whilst one group was provided distilled water (negative control). The mice were supplied with two 250 mL bottles of respective drinks, which were replaced three times per week. The pH of the cola drink was monitored over three days before the study, indicating no significant change in pH.

Two of the three experimental groups underwent topical application of either a solution containing silver diamine fluoride followed by potassium iodide (Riva Star Aqua^®^ (Technomedics Norge AS, Askim, Norway), 38% SDF 48,000 ppm F with a silver concentration of 253,900ppm and KI, pH 7.4) or Duraphat^®^ varnish (Colgate Palmolive AS. Copenhagen, Denmark) (NaF, 22,600 ppm F, pH 7.0) on the surface of their molar teeth. To apply the treatment, the mice in the two experimental groups were sedated shortly (for two minutes) with an inhalation anesthetic (Isoflurane, concentration 2.5–3.0%) [26]. One mouse at a time was firstly transferred from the cage to the induction chamber containing isoflurane gas. When the mouse was sedated, it was transferred to a bench next to the isoflurane chamber and the twitch reflex was checked. Only when there was no response to the stimuli was the application of fluoride performed. If any positive reflex response was observed, the mouse was put back into the isoflurane chamber and sedated for another one minute. Successful sedation enabled approximately 15 s of working time to apply the fluorides. The lower jaw of the animal was gently pulled down by the cheek and neck skin folds with the thumb and index finger while holding the mouse’s head and the rest of the body. This maneuver enabled access to the lower and upper teeth. The treatment was then carefully applied on the surface of the teeth with a micro brush soaked in the treatment substance. After application, the mouse was brought back to the cage and observed briefly. Only when the mouse was awake again and exhibiting the righting reflex was the following animal sedated. These procedures were performed twice a week for five weeks. After six weeks, all animals were sacrificed by cervical dislocation, and their heads were fixed in 70% ethanol.

### 2.2. Scanning Electron Microscopy

The mandibular molars were dissected and fixed in 70% ethanol. The teeth were then thoroughly cleaned by dissection and gentle brushing under running tap water, followed by air-drying overnight and mounting on brass cylinders using cyanoacrylate glue. They were sputter-coated with platinum and observed using a Tabletop Microscope TM4000Plus (Hitachi, Tokyo, Japan) operated at 10 kV.

The jaw segments were embedded in Epon and ground transversely under a stereomicroscope using 800 and 1200 grit 3M waterproof silicon carbide paper (3M, St. Paul, MN, USA). Thereafter, the ground surfaces were polished by grinding the specimens against the backside of the 3M waterproof silicon carbide paper with 0.05 µm particle seize alumina powder (Buehler Micropolish, Buehler, Lake Bluff, IL, USA) in water. Afterwards, the teeth were carefully brushed under running tap water and etched for 45 s in 1% nitric acid. Followed by air-drying overnight, they were sputter-coated with a 30 nm layer of platinum and observed using SEM. The grinding method used to achieve a transversal ground plane by SEM observation has been previously stated [25].

### 2.3. Quantifications and Statistical Analysis

SEM images of the transversely ground and etched plane were utilized to measure the lingual tooth height and lingual enamel/dentin loss. Mean values and standard deviations were calculated using Microsoft Excel Worksheet (Microsoft Excel Office Excel, 2020). The measurement data were tabulated and analyzed with the Statical Package for Social Sciences (SPSS) version 22.0 for Windows (SPSS Inc., Chicago, IL, USA). One-way analysis of variance (ANOVA) followed by the Tukey post hoc test, as well as independent *t*-tests, were employed to evaluate the data. *p*-values < 0.05 were considered statically significant.

## 3. Results

**Figure 2:**
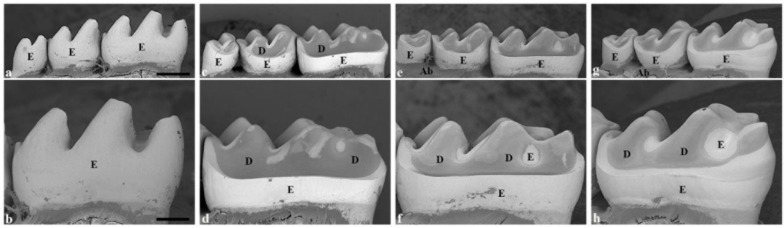
SEM images of *mandibular* molars from negative control (**a**,**b**), positive control (**c**,**d**), fluoride varnish (**e**,**f**), and silver fluoride solution (**g**,**h**) mouse. Lingual view of the molars (**a**,**c**,**e**,**g**) and higher magnification of lingual view of mandibular first molar (**b**,**d**,**f**,**h**). The bar represents 500 μm in panels (**a**,**c**,**e**,**g**) and 250 μm in panels (**b**,**d**,**f**,**h**). E = enamel, D = dentin, Ab = alveolar bone.

**Figure 3:**
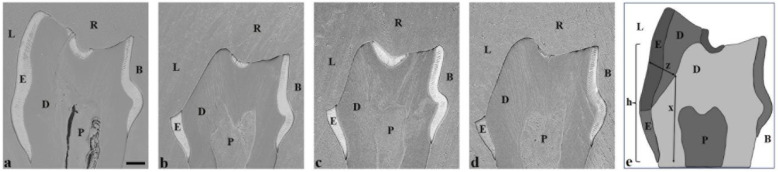
SEM images (**a**–**d**) and schematic representation (h) of transversely ground planes of mandibular first molars. SEM images of transversely ground planes of mandibular first molars from negative control (**a**), positive control (**b**), fluoride varnish (**c**), and silver fluoride solution (**d**). A schematic representation (**e**) of ground molar planes as a method to compare the experimental groups and to illustrate the measurement techniques used. Z indicates the extent of lingual enamel/dentin loss attributable to erosion. X indicates the line from enamel–cementum junction to eroded dentin surface, which is the point from where “Z” is measured. H represents the lingual tooth height, measured from the enamel–cementum junction to the highest point on the lingual aspect of the tooth. The dimensions shown correlate with the findings presented in Table 1. The bar represents 100 μm in panels (**a**–**d**). E = enamel, D = dentin, P = pulp, R = resin, B = buccal side, L = lingual side.

Comparing the negative control teeth (Figure 2a,b) with the positive control group, the teeth exposed to the cola drink exhibited a specific erosion pattern in the lingual aspects (Figure 2c,d and Figure 3b). These molars showed a more rounded, curved cuspal morphology, indicating severe erosion. A distinct erosive step was observed lingually in all experimental groups, separating the unaffected cervical region from the occlusal region of the lingual enamel (Figure 3b–d). Further, in the direction of the occlusal surface, the lingual enamel was eroded and dentin was exposed. Observation by SEM indicated that dental erosion varied to some extent between the experimental groups receiving fluoride application (Figure 2e–h and Figure 3c,d) when compared to positive control molars (Figure 2c,d and Figure 3b). However, a common finding in all experimental groups was unaffected lingual enamel, situated below this erosive step (Figure 3b–d). The most severe erosion was seen in the positive control group (Figure 2c,d and Figure 3b), whereby the enamel was completely eroded lingually, exposing the dentin. In addition, the dentin was vividly displayed on all cusps, with solely enamel remnants present in the grooves between the cusps (Figure 2c,d). The curved aspect of the lingual surface was diminished because of the erosion process in the pulpal direction (Figure 3b). Molars in the positive control compared to the negative control (Table 1) had a reduction in lingual tooth height of 40% (435 µm vs. 723 µm). In addition, the loss of lingual enamel/dentin was measured to be 136 ± 23 µm, when compared to the negative control (Figure 3e).

By observing SEM images of molars treated with silver fluoride solution, the teeth showed signs of considerable erosion with a more rounded morphology of the cusps (Figure 2g,h). The molars exhibited more enamel remnants and a more curved lingual outline compared to teeth which had the fluoride varnish applied (Figure 2g,h and Figure 3d). In the group treated with the silver fluoride solution, the lingual tooth height was reduced by 29% (510 µm vs. 723 µm) compared to the negative control (Table 1). Additionally, the lingual enamel/dentin loss in the silver fluoride solution-treated molars measured 88 ± 17 µm (Table 1), giving a 35% reduction in tooth tissue loss compared to the untreated positive control molars (Table 1).

The application of fluoride varnish on molar teeth resulted in less erosion compared to the positive control group (Table 1). From the lingual aspect, the enamel was eroded greatly; however, between the cusps in the grooves, there existed marginally more enamel than the positive control (Figure 2e,f). Following the erosion process towards the pulp, the characteristic curved outline of the lingual surface was diminished (Figure 3c). Molars which had fluoride varnish applied showed a reduction in lingual tooth height by 35% (472 µm vs. 723 µm) compared to the negative control (Table 1). Further, the loss of lingual/dentin loss measured 129 ± 35 µm (Table 1), indicating a reduction in tooth tissue loss of 5% compared to untreated positive control molars (Table 1).

**Figure 4:**
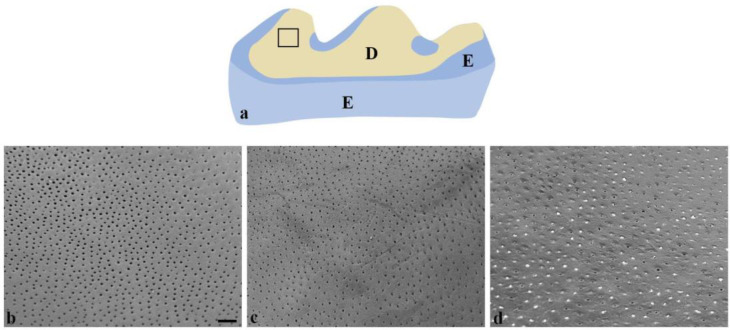
Schematic representation of (**a**) eroded lingual aspect of the mandibular first molar and SEM images (**b**–**d**) of exposed dentin tubules. SEM images of exposed dentin tubules of mandibular first molars from positive control (**b**), fluoride varnish (**c**), and silver fluoride solution (**d**). A schematic representation of the first molar from the lingual side is presented to indicate the area for SEM imaging (**a**). The bar represents 10 μm in panels (**b**–**d**). E = enamel, D = dentin.

Figure 4a is a schematical representation of the lingual aspect of the first mandibular molar, on which the black square indicates the areas Figure 4b–d are extracted from. The positive control group (Figure 4b) showed open and enlarged dentinal tubules, with little visible peritubular dentin. In comparison, the group treated with fluoride varnish (Figure 4c) exhibited a dentin surface with still open but narrowed tubules (Figure 4b). Lastly, the mandibular molars treated with silver fluoride solution demonstrated several occluded dentinal tubules caused by the formation of a white precipitate and a precipitated layer at the intertubular dentin (Figure 4d).

## 4. Discussion

To minimize the risk of developing dental erosion, the natural approach is to reduce the exposure of the oral cavity to acid. However, where this is deemed difficult to control, optimum preventive treatments should be implemented [13], such as use of fluoride. The erosion-inhibiting effect of fluorides results in the accumulation of CaF_2_ on the surface, serving as a reservoir of calcium ions to neutralize hydrogen ions from acids or by forming fluorid hydroxyapatitt [27].

The present study explored the effect of a silver fluoride solution believed to have diverse effects, particularly the ability to arrest caries progression and to limit the development of new cavities [28]. This silver fluoride solution is without ammonia and the pH is lower than other SDF products: pH 7.4 compared to pH 9–10 [29]. The solution emits less odour and metallic taste but has the same efficacy as SDF with ammonia [30]. In the present study, the application of a solution consisting of silver fluoride (Figure 2 and Figure 3) resulted in a significant protective effect on the development of dental erosion compared to the positive control group. Additionally, there was a clear presence of more enamel structure and a higher degree of intact dentin in the silver fluoride solution-treated teeth group when comparing the silver fluoride solution to the fluoride varnish.

At higher magnification (×800) of the dentin surfaces, the positive control group showed wider dentin tubules with more eroded peritubular dentin. This is in line with the higher mineral content in the peritubular dentin compared to inter-tubular dentin and hence a greater loss of minerals in dentin tubules. However, SEM images with silver fluoride solution-treated surfaces displayed occluded dentinal tubules, visualized as white remnants or plugs covering the openings of the tubules and a precipitated layer at intertubular dentin (Figure 4d). This observation is consistent with other research studies of SDF solution [31,32,33]. Moreover, the formation of different silver compounds with high stability and insolubility, such as silver Ag_3_PO_4_, AgCl, and AgF, has been seen [31]. SDF and KI together have been described to form insoluble silver iodide which occludes the dentinal tubules and immediately eliminates dental hypersensitivity [30].

Silver compounds are thought to form a stable, acid-resistant layer and act as a physical barrier on the tooth surface [31]. Hence, the current results are both highly important in the treatment of dentin hypersensitivity and in line with the hydrodynamic theory [34]. Furthermore, the protective effect of SDF has also been linked to the inhibition of collagenase and MMPs, preserving the collagen matrix of the intertubular dentin [35]. In the case where MMPs are active, the enzymes contribute to the removal of the organic matrix, and the demineralization process is thought to increase [36]. Accordingly, preserving the organic matrix is thought to stop ionic diffusion after an acidic challenge, and the progression of dental erosion is therefore restrained [36]. The somewhat deepened position of the dentin tubules, compared to the level of intertubular dentin (Figure 4d), may be due to this preservation effect, and it is found to be less pronounced in both fluoride varnish-treated dentin (Figure 4c) and the positive control (Figure 4b).

There has been observed some side effects with the application of the SDF solution. The most profound effect is staining of the treated tooth surfaces. However, with the application of KI after an SDF solution, the staining effect is thought to be reduced. According to a study describing the prevention of dental erosion in primary teeth and the staining potential of SDF-KI, CPP-ACPF (casein phosphopeptide-amorphous calcium phosphate fluoride), NaF (sodium fluoride) varnishes, and SDF, SDF-KI was as effective as the others, and there was no statistically significant effect regarding its staining [37]. This is of great importance, indicating that this silver fluoride solution is a preferable solution in various treatment scenarios.

In contrast, fluoride varnish treatment resulted in 5% reduction in tooth tissue loss compared to the positive control group. This may be related to the fact that the active substance NaF needs a long application period, low pH, and a high concentration to achieve the best effect [38]. However, there are conflicting results concerning the level of protective effect found in traditional fluorides on dental erosion [14].

In this study, two high fluoridated commercial products with a different viscosity, composition, and fluoride concentration were included. Limited research exists regarding the current silver fluoride solution; however, the preventive effects are considered similar to a solution of SDF [30]. The polyvalent metal compound can promote remineralization, preventing demineralization and collagen degradation, including the closure of the dentinal tubules [39]. In line with our research, the silver component interacts with the teeth’s minerals and forms different silver ions and silver-containing hydroxyapatite [40]. This integration into the tooth is important and unique in the preventive work against dental erosion. On the other hand, the fluoride varnish forms a CaF_2_ layer, i.e., short-lived after the exposure to an acidic environment [14]. By this means, the use of conventional fluorides regarding dental erosion is limited.

To the best of our knowledge, this is the first in vivo study exploring dental erosion and the protective effects of fluoride varnish and silver fluoride solution. The typical pattern of erosion as currently described has previously been observed in both rats [41,42,43] and mice [25,44]. A research study utilizing an animal model, especially mice, resembles the human milieu orally, and it is, in general, a good method for studying oral pathology. The dentition of the mouse and human display considerable resemblance in terms of tooth development, including the presence of the same underlying molecular networks [45]. Additionally, the structural components of the enamel, the prism, and interprism are the same in humans and mice. However, the positioning of the elements, the pattern of the prisms, is not identical [46]. In current study, we engaged a standardized, well controlled in vivo model, which is detailed and has been used in our previous research. This method is highly successful for investigating dental erosion and the initial stages of erosive lesions [25].

The observation of the lingually intact enamel under the erosive step supports previous findings [25]. We suspect this to be a result of gingiva’s ability to conceal and protect this cervical region and possibly an effect from the gingival crevicular fluid. Interestingly, the buccal aspect of the teeth was not affected by the acid. The fact that the grave erosion was only seen lingually may be linked to the rapid movement of the mouse tongue, the retention of acid in the tongue papillae or the somewhat less pronounced rodent labial groove towards the corners of the mouth [47,48]. Previous investigations have confirmed that the movement of the tongue can escalate dental erosion [49]. A feasible speculation is the consequence of a powerful musculus masseter, continuously working as a rodent, hiding the buccal side and minimizing the erosion process.

In this work, the transversal ground planes of the first mandibular molar enabled precise measurements of tooth erosion (Figure 3). However, the technical difficulties with achieving an ideal transversal ground plane through the correct cusps regarding the small size of the dentition might influence the exactness of the measurements [25] without affecting the overall significant differences in the tooth height and enamel or the dentin loss measured in each group. This mouse model is well-suited for future research on various fluoride compounds and their effects on dental erosion. It is important to acknowledge that this is an explorative study under highly controlled and extreme acidic conditions. In addition, the continuous consumption of acidic drinks, performed over several weeks, is seldom seen in human circumstances.

## 5. Conclusions

- This current study explores the prophylactic effects of a metal fluoride solution, silver fluoride solution, in comparison to a traditional fluoride varnish in a mouse model.
- The silver fluoride solution indicates the best effect by reducing the exposure of dentin when compared to fluoride varnish.
- Silver fluoride solution may occlude the dentinal tubules, giving the solution an important role in the preventive work against dentin hypersensitivity.

## Figures and Tables

**Table 1:** Dimensions of lingual tooth height and enamel/dentin loss.

	Negative ctr.	Positive ctr.	Fluoride Varnish	Silver Fluoride Solution
Lingual tooth height (h)	723 ± 29	435 ± 42	472 ± 34	510 ± 37 *
Lingual enamel/dentin loss (z)	-	136 ± 23	129 ± 35	88 ± 17 *

The values represent measurements taken from SEM images of transversely ground planes, as shown in Figure 3. The measured dimensions (mean ± SD, μm) of mandibular first molar lingual tooth height and enamel/dentin loss are presented. Letters in parentheses (h and z) refer to the letters in Figure 3e. (-) Not applicable. (*) Significant difference, *p* < 0.05, when compared to positive control.

## Data Availability

The original contributions presented in the study are included in the article; further inquiries can be directed to the corresponding author.
